# Karatsuba Algorithm Revisited for 2D Convolution Computation Optimization

**DOI:** 10.3390/e27050506

**Published:** 2025-05-08

**Authors:** Qi Wang, Jianghan Zhu, Can He, Shihang Wang, Xingbo Wang, Yuan Ren, Terry Tao Ye

**Affiliations:** 1 The Department of Electrical and Computer Engineering, University of British Columbia, Vancouver, BC V6T 1Z4, Canada; 2 The Department of Electrical and Electronic Engineering, Southern University of Science and Technology, Shenzhen 518055, China; 3 The Department of Electrical and Electronic Engineering, The University of Hong Kong, Pokfulam Road, Hong Kong; 4 School of Science and Engineering, The Chinese University of Hong Kong, Shenzhen 518172, China; 5 Institute of Nanoscience and Applications, Southern University of Science and Technology, Shenzhen 518055, China; 6 Jiaxing Research Institute, Southern University of Science and Technology, Jiaxing 518055, China

**Keywords:** Karatsuba algorithm, convolutional computing complexity, hardware computation acceleration, Winograd algorithm, hardware/software co-design

## Abstract

Convolution plays a significant role in many scientific and technological computations, such as artificial intelligence and signal processing. Convolutional computations consist of many dot-product operations (multiplication–accumulation, or MAC), for which the Winograd algorithm is currently the most widely used method to reduce the number of MACs. The Karatsuba algorithm, since its introduction in the 1960s, has been traditionally used as a fast arithmetic method to perform multiplication between large-bit-width operands. It had not been exploited to accelerate 2D convolution computations before. In this paper, we revisited the Karatsuba algorithm and exploited it to reduce the number of MACs in 2D convolutions. The matrices are first segmented into tiles in a divide-and-conquer method, and the resulting submatrices are overlapped to construct the final output matrix. Our analysis and benchmarks have shown that for convolution operations of the same dimensions, the Karatsuba algorithm requires the same number of multiplications but fewer additions as compared with the Winograd algorithm. A pseudocode implementation is also provided to demonstrate the complexity reduction in Karatsuba-based convolution. FPGA implementation of Karatsuba-based convolution also achieves 33.6% LUTs (Look -up Tables) reduction compared with Winograd-based implementation.

## 1. Introduction

Various methods have been proposed to reduce the computational complexity, in particular, the number of multiplication–accumulation (MAC) operations needed to perform matrix convolution operations. Among these methods, Fast Fourier Transform (FFT) [1,2,3] and Winograd algorithm [4,5] are the two most widely used ones. They are now incorporated into many popular AI computational frameworks to optimize and accelerate convolution calculations.

FFT-based convolution optimization can significantly reduce computational complexity, but this technique is only effective when the kernel matrix size is large enough, i.e., 7 by 7 or even larger [1,2]. As demonstrated by Mathieu et al. [2] and Vasilache et al. [3], FFT-based methods transform convolution operations into element-wise multiplications in the frequency domain, achieving asymptotic complexity reduction. However, most filter kernels in today’s AI and image processing applications use small-sized matrices, which are not applicable for FFT-based optimization. Chi et al. [6] have recently proposed Fast Fourier Convolution (FFC) to address some limitations of traditional FFT approaches by incorporating non-local receptive fields, but it does not help to reduce the computation complexity with small kernels remaining.

The Winograd algorithm [4] is another widely used technique to reduce the complexity of matrix convolution. Instead of direct dot-product calculation, the Winograd algorithm first transforms the input array into matrices of higher dimensions and then precalculates the intermediate terms that can be later reused by different dot-products. Through this operation, the number of multiplications can be effectively reduced [4,5]. Lavin and Gray [5] demonstrated that Winograd’s minimal filtering algorithms provide substantial speedups for convolutions with small filter sizes, making them particularly suitable for modern convolutional neural networks. However, as noted by Meng and Brothers [7], Winograd methods still face challenges with numerical stability and require complex transformation matrices.

Some works have implemented efficient convolution operators, such as the im2win [8,9], which leverages the data locality characteristics of convolution. By reorganizing the input tensor, im2win achieves reduced memory consumption and improved data access efficiency. However, this method does not reduce the computational complexity or decrease the number of MAC operations; it merely enhances performance through optimized memory access patterns.

In this paper, we exploit the Karatsuba algorithm to further reduce the complexity of convolution computation. The Karatsuba algorithm was originally proposed in the 1960s by Anatolii Karatsuba [10] to compute the multiplication of operands with large bit-width by splitting the operands into vectors of smaller-sized bit-width [11,12]. Heideman [13] states that convolution is essentially equivalent to polynomial multiplication, so the Karatsuba algorithm can be applied to convolution. Some previous works [14] have explored the Karatsuba algorithm for one-dimensional convolution. However, to the best of our knowledge, its application to 2D convolution optimization as an operand remains unexplored. Our approach differs fundamentally from both FFT and Winograd methods: unlike FFT, which operates in the frequency domain and struggles with small kernels, and unlike Winograd, which requires complex transformation matrices, our Karatsuba-based approach works directly with spatial domain representations while reducing computational complexity.

In our exploitation, through the sharing of the vectors of adjacent elements in the kernel matrix, also referred to as a filter or weight matrix, we can effectively reduce the number of MACs as compared with the implementation from the direct dot-product as well as the Winograd algorithm. To be more specific, for the convolution between kernel matrix and input matrix, the Karatsuba algorithm uses the same number of multiplications as compared with the Winograd algorithm (using the same kernel and input matrices), but with a much smaller number of additions. This reduction in additions is particularly significant for hardware implementations.

A pseudocode that implements Karatsuba-based 2D convolution is provided in this paper as an appendix. To demonstrate its potential in reducing hardware resources, an FPGA implementation of matrix convolution based on the Karatsuba algorithm is also benchmarked. The results demonstrate its advantages in the reduction in the logic gate count (Look-up Table, or LUT) by up to 33.6% compared with Winograd-based convolution implementation (also in FPGA).

The rest of this paper is structured as follows: Section 2 introduces the basis of the Winograd and Karatsuba algorithms and their usage in multiplication optimization. Section 3 further demonstrates how Karatsuba can be implemented in the kernel matrix multiplication in convolution. Section 4 quantitatively evaluates the number of operations, including multiplications and additions, required in the Karatsuba algorithm and compares the operation counts with those of the Winograd algorithm. An FPGA-based hardware implementation is described in Section 5 and its performance is analyzed. The conclusion is given in Section 6.

## 2. Background

### 2.1. Traditional Dot-Product Convolution Computation

Typical convolution is the operation between two operands, i.e., the input feature map matrix, and the kernel matrix (also referred to as the filter, or the weight parameter matrix).

The input feature map is a multidimensional matrix, which can be characterized by its height, width, and channel or *x*, *y*, and *c*, respectively. The other operand, the filter or kernel, is the matrix with the same width and height, *u* and *v* (in many cases u=v), and normally also with the same number of channels *c* as the input feature map [15].

The convolution between the kernel matrix and the feature map is performed through a dot-product operation and can be described as follows: The kernel (sized c,u,v) overlays on top of the input feature map (sized c,x,y). The elements at the corresponding position of the input feature map and the kernel multiply, and their products are summed as one output (dot-product). The kernel matrix slides through the feature map for each element, and the dot-product of every sliding step forms the output matrix with the size (c,x−u+1,y−v+1) [16].

**Figure 1:**
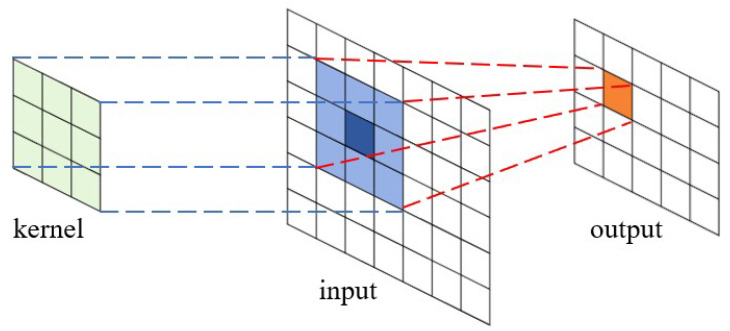
Convolution operation between a kernel matrix and an input feature map (matrix), and the resulting output matrix. The kernel matrix overlaps with the input matrix, and the corresponding elements in the two matrices perform the dot-product operations. The products are summed up to calculate the elements in the output matrix.

If the kernel filter is denoted as $G_{c,m,n}$, the input feature is denoted as $D_{c,x,y}$, and the output matrix $Y_{x,y}$ can be calculated as:

(1) $$Y_{x,y}=\sum_{c=1}^{C}\sum_{m=1}^{u}\sum_{n=1}^{v}D_{c,x+m,y+n}G_{c,m,n}$$

where *x* and *y* are the coordinates of the feature map tiles, and *m*, *n*, and *c* are three iterators on different dimensions of the kernel. The process is shown in Figure 1.

The above equation (Equation (1)) demonstrates that the convolution operation actually consists of lots of multiplication–accumulation (MAC) operations. Actually, MAC operations account for the majority of the convolution workload. Particularly, multiplications need many more logic gates to perform the arithmetic operations than additions, and the required hardware resources will increase almost quadratically with the increase in the bit-width of the operands. Table 1 lists the logic LUTs needed in an FPGA platform to implement the multiplication and addition operations with operands of different bit-width. The number of logic LUTs correlates to the complexity of hardware implementation, which will also correlate to the power consumption as well as other hardware overheads.

### 2.2. Winograd Algorithm

The Winograd minimal filtering algorithm is a popular method to optimize the convolution computation when the kernel size is small [5]. The algorithm can be simply explained through the following example.

A convolution between a 4-element input vector $d=(d_{0},d_{1},d_{2},d_{3})$ and 3-element filter $g=(g_{0},g_{1},g_{2})$ to produce a 2-element output can be denoted as F(2,3) (indicating 2 output elements and 3 filter elements). The input array *d* is first folded into a two-dimensional matrix $\left[\begin{array}{ccc} d_{0} & d_{1} & d_{2}\\ d_{1} & d_{2} & d_{3} \end{array}\right]$, and the convolution operation can be transformed into the multiplication of two matrices, as shown in the following equations:

(2) $$F\left(2,3\right)=\left[\begin{array}{ccc} d_{0} & d_{1} & d_{2}\\ d_{1} & d_{2} & d_{3} \end{array}\right]\left[\begin{array}{c} g_{0}\\ g_{1}\\ g_{2} \end{array}\right]=\left[\begin{array}{c} m_{0}+m_{1}+m_{2}\\ m_{1}-m_{2}-m_{3} \end{array}\right]$$

where

(3) $$m_{0}=\left(d_{0}-d_{2}\right)g_{0}\;m_{1}=\left(d_{1}+d_{2}\right)\frac{g_{0}+g_{1}+g_{2}}{2}$$

(4) $$m_{3}=\left(d_{1}-d_{3}\right)g_{2}\;m_{2}=\left(d_{2}-d_{1}\right)\frac{g_{0}-g_{1}+g_{2}}{2}$$

Since $\frac{g_{0}+g_{1}+g_{2}}{2}$ and $\frac{g_{0}-g_{1}+g_{2}}{2}$ are common terms for all inputs and are precalculated, through these transformations, the convolution requires 4 multiplications and 8 additions (without counting the common terms).

The calculation process of the Winograd algorithm can be generalized into three steps: (1) Preprocessing, which is to transform the input data and the filter into the folded matrix format, such as $\left(d_{0},d_{1},d_{2}\right)$ and $\left(d_{1},d_{2},d_{3}\right)$ in the previous case. (2) Calculation of the intermediate terms, using the equations from Equation (3) and (4). (3) A combination of the intermediate terms and derive the final value.

For the convenience of calculation, Winograd convolution can be represented by the following matrix operations [5]:

(5) $$Y=A^{T}\left[\left(Gg\right)⊙\left(B^{T}d\right)\right]$$

In this equation, ⊙ indicates element-wise multiplication. *d* and *g* are input data vector and filter vector, respectively. A,B, and *G* are the transformation matrix of *d* and *g*. For operation F(2,3), A,B, and *G* are defined as follows:

(6) $$\begin{array}{cc} A & ={\left[\begin{array}{cccc} 1 & 1 & 1 & 0\\ 0 & 1 & -1 & -1 \end{array}\right]}^{T}\\ B & ={\left[\begin{array}{cccc} 1 & 0 & -1 & 0\\ 0 & 1 & 1 & 0\\ 0 & -1 & 1 & 0\\ 0 & 1 & 0 & -1 \end{array}\right]}^{T}\\ G & =\left[\begin{array}{ccc} 1 & 0 & 0\\ \frac{1}{2} & \frac{1}{2} & \frac{1}{2}\\ \frac{1}{2} & -\frac{1}{2} & \frac{1}{2}\\ 0 & 0 & 1 \end{array}\right] \end{array}$$

In general, for an operation with *m* output-elements and *r* filter-elements in one dimension, the Winograd operation can be denoted as F(m,r), only m+r−1 multiplications are needed [17].

For a two-dimensional Winograd, its calculation formula can be deduced from Equation (5), especially, in the case of two-dimensional inputs, i.e., Winograd F(2×2,3×3) is the nesting operation of F(2,3). Operation matrices in A,B,G in Equation (5) are replaced with the two-dimensional forms, as expressed in Equation (7):

(7) $$Y=A^{T}\left[\left(GgG^{T}\right)⊙\left(B^{T}dB\right)\right]A$$

A pseudocode to calculate F(2×2,3×3) using the Winograd algorithm is listed in the Appendix A section of this paper, along with a detailed counting of the number of multiplications and additions needed to perform the operation. To be more specific, F(2×2,3×3) needs 16 multiplications and 77 additions. In other words, Winograd needs 4 multiplications and 19.25 additions for each output element (output is a 2 × 2 matrix and has 4 elements). The traditional direct dot-multiplication requires 36 multiplications and 24 additions for 2×2 outputs, which means 9 multiplications and 6 additions for each output.

In general, for the convolution operation with the filter *g* of size r×r, the input matrix *d* of size (m+r−1)×(m+r−1), and the output matrix of size m×m, using Winograd optimization, the hardware complexity (in terms of number of multiplications for each output element) is given by $\frac{{\left(m+r-1\right)}^{2}}{m^{2}}$ [17]. As the filter size increases, the multiplicative complexity per output element will decrease. However, the number of additions will increase. Because the hardware overhead for multiplication is much higher than that of the addition, especially for the operations with larger bit-width, the saving from the multiplication reduction will always more than offset the increase from addition. And Winograd optimization will always outperform the direct matrix dot-multiplication in terms of hardware overheads and computation latency.

### 2.3. Karatsuba Algorithm

The Karatsuba algorithm, also known as the Karatsuba–Ofman algorithm, was proposed in the 1960s to optimize the calculation of the multiplication between operands with large bit-width, i.e., the bit-width is larger than the bit-width of the CPUs or MCUs, and the product cannot be computed directly. The Karatsuba algorithm partitions the larger bit-width operands into several smaller bit-width operands, the products of the smaller bit-width operands are calculated in a pair-wise manner and summed up by additions and shiftings [18,19].

The divide-and-conquer steps of the Karatsuba algorithm can be more accurately described as follows.

Assume *A* and *B* are two 2n-bit operands and can be evenly divided into two smaller operands, i.e., $a_{1},a_{2}$ or $b_{1},b_{2}$, where $a_{1}$ is the LSB (Least Significant Bit) part while $a_{2}$ is the MSB (Most Significant Bit) part, and $X=2^{n}$, as described in Equation (8).

(8) $$A=a_{1}+a_{2}X,\;B=b_{1}+b_{2}X$$

The multiplication between operands *A* and *B* can be rewritten as Equation (9)

(9) $$A\times B=a_{2}b_{2}X^{2}+\left(a_{2}b_{1}+a_{1}b_{2}\right)X+a_{1}b_{1}$$

Equation (8) converts the 2n-bit multiplication into four n-bit multiplications. This conversion is called the divide-and-conquer process. In a direct multiplication process, a total of four cross-term products are needed, and these four products can be calculated in parallel. In order to reduce the number of multiplications, the Karatsuba algorithm introduces another cross-term $\left(a_{2}+a_{1}\right)\times \left(b_{2}+b_{1}\right)$, as written in Equation (10).

(10) $$\left(a_{2}b_{1}+a_{1}b_{2}\right)=\left(a_{2}+a_{1}\right)\times \left(b_{2}+b_{1}\right)-a_{2}b_{2}-a_{1}b_{1}$$

Then, the A×B product can be transformed into

(11) $$\begin{array}{cc} A\;\times \;B\; & =\;a_{2}b_{2}X^{2}\;+\;\left[\left(a_{2}\;+\;a_{1}\right)\;\times \;\left(b_{2}\;+\;b_{1}\right)\;-\;a_{2}b_{2}\;-\;a_{1}b_{1}\right]X\;+\;a_{1}b_{1}\\ \; & =a_{2}b_{2}\left(X^{2}\;-\;X\right)\;+\;\left(a_{2}\;+\;a_{1}\right)\;\times \;\left(b_{2}\;+\;b_{1}\;\right)\;X\;\;+\;\;a_{1}b_{1}\;\left(\;1\;-\;X\;\right) \end{array}$$

Equation (11) has three product terms, i.e., $a_{1}b_{1},a_{2}b_{2}$ and $\left(a_{1}+a_{2}\right)\times \left(b_{1}+b_{2}\right)$. $a_{1}b_{1}$ and $a_{2}b_{2}$ are reused in the calculation of the terms $\left(a_{2}b_{1}+a_{1}b_{2}\right)$. Only three multiplications are needed to obtain the same result, which means one multiplication has been saved. The saving comes with the expense that several new additions and shiftings have been added to the computation process. However, from Table 1 we can see that additions take up much less computing resources than multiplications, so the reduction in multiplications will offset the extra computation resources caused by the additional additions/shiftings.

A similar divide-and-conquer process can be constructed to divide the long bit-width operands into three terms, i.e.,

(12) $$A=a_{1}+a_{2}X+a_{3}X^{2},\;B=b_{1}+b_{2}X+b_{3}X^{2}$$

where $a_{1},a_{2}$, and $a_{3},b_{1},b_{2}$ and $b_{3}$ are the three evenly divided terms from LSB to MSB of operands *A* and *B*, respectively, the direct product between *A* and *B* needs nine multiplications, as shown in Equation (13),

(13) $$\begin{array}{cc} A\times B & =a_{3}b_{3}X^{4}+\left(a_{3}b_{2}+a_{2}b_{3}\right)X^{3}+(a_{3}b_{1}+a_{2}b_{2}\\ \; & +a_{1}b_{3})X^{2}+\left(a_{1}b_{2}+a_{2}b_{1}\right)X+a_{1}b_{1} \end{array}$$

Actually, the Karatsuba process allows several different reorganization methods to reduce the number of multiplications. The following equations represent one of the optimizing methods, where the cross-terms in Equation (13) can be rewritten as;

(14) $$\begin{array}{cc} (a_{3}b_{2}\;+\;a_{2}b_{3})\; & =\;\left(a_{3}\;+\;a_{2}\right)\;\times \;\left(b_{3}\;+\;b_{2}\right)\;-\;a_{3}b_{3}\;-\;a_{2}b_{2} \end{array}$$

(15) $$\begin{array}{cc} (a_{2}b_{1}\;+\;a_{1}b_{2})\; & =\;\left(a_{2}\;+\;a_{1}\right)\;\times \;\left(b_{2}\;+\;b_{1}\right)\;-\;a_{2}b_{2}\;-\;a_{1}b_{1} \end{array}$$

(16) $$\begin{array}{cc} (a_{3}b_{1}\;+\;a_{1}b_{3})\; & =\;\left(a_{3}\;+\;a_{1}\right)\;\times \;\left(b_{3}\;+\;b_{1}\right)\;-\;a_{3}b_{3}\;-\;a_{1}b_{1} \end{array}$$

Replacing these terms in Equation (13), the 9 multiplications can be reduced to 6 multiplications.

(17) $$\begin{array}{cc} A\times B & =a_{3}b_{3}X^{4}+\left(a_{3}b_{2}+a_{2}b_{3}\right)X^{3}+(a_{3}b_{1}+a_{2}b_{2}\\ \; & \;+a_{1}b_{3})X^{2}+\left(a_{1}b_{2}+a_{2}b_{1}\right)X+a_{1}b_{1}\\ \; & =a_{3}b_{3}\left(X^{4}-X^{3}-X^{2}\right)\\ \; & +a_{2}b_{2}\left(X^{2}-X^{3}-X^{1}\right)\\ \; & +a_{1}b_{1}\left(X^{0}-X^{2}-X^{1}\right)\\ \; & +\left(a_{3}+a_{2}\right)\times \left(b_{3}+b_{2}\right)X^{3}\\ \; & +\left(a_{3}+a_{1}\right)\times \left(b_{3}+b_{1}\right)X^{2}\\ \; & +\left(a_{2}+a_{1}\right)\times \left(b_{2}+b_{1}\right)X^{1} \end{array}$$

Actually, the Karatsuba algorithm can be applied to split the long bit-width operands into any number of segments and apply similar techniques to reduce the number of multiplications to calculate the final product.

## 3. Applying the Karatsuba Algorithm in Convolution

### 3.1. A Simple Example

We first describe the Karatsuba algorithm through a simple example and later generalize the operation to all cases.

Convolution computation is to calculate the dot-products of the corresponding elements between the kernel matrix and the input matrix. In a direct convolution calculation, if the kernel matrix has size r×r with $r^{2}$ elements, then total $r^{2}$ multiplications are needed.

**Figure 2:**
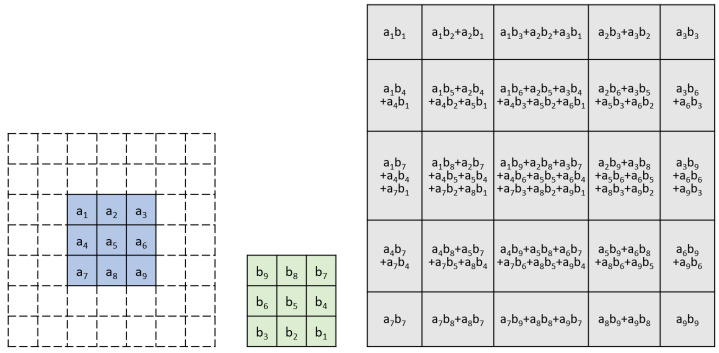
An Example of Input, Weight and Output Matrices in Karatsuba Convolution.

Assume we have a kernel matrix of 3×3 to convolute with a small input matrix of the size of 3×3, where elements in the kernel matrix are named $b_{1},b_{2}$… to $b_{9}$, and the elements in the input matrix are named as $a_{1},a_{2}$ … to $a_{9}$, respectively. For convenience of convolution, without loss of generality, we add a 2-layer padding ring around the 3×3 elements of the input matrix, and the elements in the padding ring are set to 0, shown as the blank elements in Figure 2.

**Figure 3:**
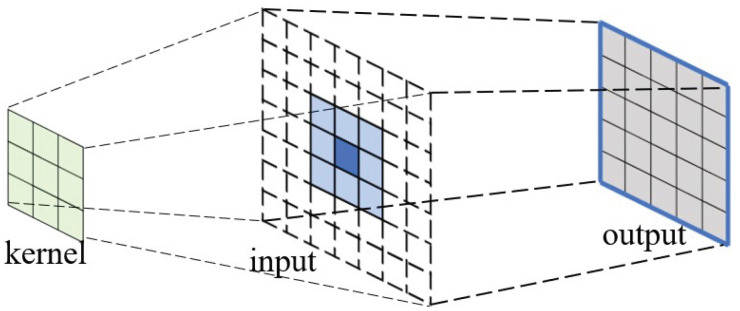
Convolution kernel, input tile, and output tile of the Karatsuba convolution algorithm. The dashed elements of the input matrix are the padding rings.

Convolution is performed by sliding the kernel matrix along the input matrix with a step of 1 element in each row and column, as shown in Figure 3. The resulting output matrix is illustrated in Figure 2.

**Figure 4:**
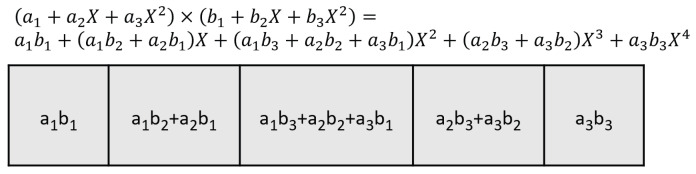
The first row of the output matrix, the elements in the row are identical to the coefficient of the Equation (13).

If we take the first row of the output matrix (shown in Figure 4) and compare the elements in the row with those terms in Equation (13), we see that they are identical with the 9 cross-terms from $\left(a_{1}+a_{2}X+a_{3}X^{2}\right)\times \left(b_{1}+b_{2}X+b_{3}X^{2}\right)$. Using the Karatsuba algorithm, the 9 cross-terms can be calculated from 6 multiplications, as shown in Equations (14)–(16).

**Figure 5:**
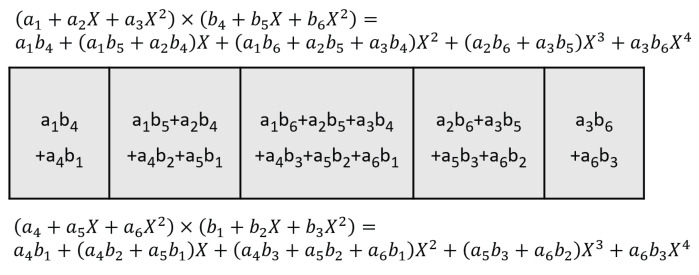
The second row of the Figure 2, the values inside the blocks can be split into the upper 9 elements and the lower 9 elements.

For the second row in the output feature map, there are 18 elements. We can split them into two groups, 9 elements in the upper group and 9 elements in the lower group, as shown in Figure 5 below.

Conversely, the upper 9 elements correspond to the cross-terms from $\left(a_{1}+a_{2}X+a_{3}X^{2}\right)\times \left(b_{4}+b_{5}X+b_{6}X^{2}\right)$, while the lower 9 elements correspond to the cross-terms from $\left(a_{4}+a_{5}X+a_{6}X^{2}\right)\times \left(b_{1}+b_{2}X+b_{3}X^{2}\right)$. The Karatsuba algorithm can be applied to calculate the 18 terms with only 12 multiplications. Similarly, the calculation of the elements in the third row, the fourth row, and the fifth row can be optimized. The total number of multiplications needed can be reduced from 81 to 54 multiplications.

The same reduction can also be performed column-wise. If we look at the first column on the left of the output matrix, we can see that the elements are identical to the cross-terms from $\left(a_{1}+a_{4}X+a_{7}X^{2}\right)\times \left(b_{1}+b_{4}X+b_{7}X^{2}\right)$. Similarly, the elements in each column are identical to the cross-terms of the multiplication of their corresponding polynomials.

When the Karatsuba algorithm is applied in either row-wise or column-wise manner, the multiplications that are needed to calculate the elements can be reduced to 2/3 of the original cross-terms.

### 3.2. Applying the Karatsuba Algorithm in Both Rows and Columns

Actually the number of multiplications for calculating the output elements can be further reduced. As the Karatsuba algorithm can be applied in both row and column simultaneously and recursively, the required multiplications can be further reduced to ${\left(2/3\right)}^{2}$, or 4/9, i.e., only 36 multiplications are needed to calculate all the 81 cross-terms in the output matrix. More than half of the multiplications can be saved.

We denote the input matrix as A and the kernel matrix as B, respectively. As introduced in the previous subsection, each row of the input matrix can be represented as a polynomial, i.e., the three rows of the input matrix can be represented as

(18) $$\begin{array}{cc} A_{1} & =a_{1}+a_{2}X+a_{3}X^{2},\\ A_{2} & =a_{4}+a_{5}X+a_{6}X^{2},\\ A_{3} & =a_{7}+a_{8}X+a_{9}X^{2} \end{array}$$

Similarly, the rows of the kernel matrix can be represented as

(19) $$\begin{array}{cc} B_{1} & =b_{1}+b_{2}X+b_{3}X^{2},\\ B_{2} & =b_{4}+b_{5}X+b_{6}X^{2},\\ B_{3} & =b_{7}+b_{8}X+b_{9}X^{2} \end{array}$$

where polynomials $A_{1},A_{2},A_{3}$, and $B_{1},B_{2},B_{3}$ represent each row of the input matrix A and kernel matrix B, respectively.

We can further combine the three polynomials $A_{1},A_{2},A_{3}$ of input matrix A into one single polynomial,

(20) $$A=A_{1}+A_{2}Y+A_{3}Y^{2},$$

And similarly, the kernel matrix B can be represented as a single polynomial

(21) $$B=B_{1}+B_{2}Y+B_{3}Y^{2},$$

where $Y=X^{3}$, indicating the shifting of the radix positions of each term in the polynomials.

After the convolution between matrix A and B, the elements in the resulting matrix correspond to the cross-terms from the multiplication between the two polynomials A×B,

(22) $$\begin{array}{cc} A\times B & =A_{3}B_{3}Y^{4}+\left(A_{3}B_{2}+A_{2}B_{3}\right)Y^{3}+(A_{3}B_{1}+A_{2}B_{2}\\ \; & \;+A_{1}B_{3})Y^{2}+\left(A_{1}B_{2}+A_{2}B_{1}\right)Y+A_{1}B_{1}\\ \; & =A_{3}B_{3}\left(Y^{4}-Y^{3}-Y^{2}\right)\\ \; & +A_{2}B_{2}\left(Y^{2}-Y^{3}-Y^{1}\right)\\ \; & +A_{1}B_{1}\left(Y^{0}-Y^{2}-Y^{1}\right)\\ \; & +\left(A_{3}+A_{2}\right)\times \left(B_{3}+B_{2}\right)Y^{3}\\ \; & +\left(A_{3}+A_{1}\right)\times \left(B_{3}+B_{1}\right)Y^{2}\\ \; & +\left(A_{2}+A_{1}\right)\times \left(B_{2}+B_{1}\right)Y^{1} \end{array}$$

As shown in Equation (22), the multiplication A×B involves six polynomial multiplications, namely, $A_{3}\times B_{3},A_{2}\times B_{2},A_{1}\times B_{1},\left(A_{3}+A_{2}\right)\times \left(B_{3}+B_{2}\right),\left(A_{3}+A_{1}\right)\times \left(B_{3}+B_{1}\right)$, and $\left(A_{2}+A_{1}\right)\times \left(B_{2}+B_{1}\right)$. As discussed previously, each of the polynomial multiplication need 6 multiplications by using the Karatsuba algorithm, so in total, we only need 36 multiplications to calculate all the elements in the convolution, as compared with 81 from direct dot-product calculation.

We also provide pseudocodes that implement the convolution between a 3×3 kernel matrix and a 3×3 input matrix using the Karatsuba algorithm, and the number of multiplications and additions is also counted. In summary, only 36 multiplications are needed along with 120 additions. The details of the pseudocodes and the operation counts are listed in the Appendix B Section.

### 3.3. Convolution on a Large Input Matrix

The above example is actually a convolution between a 3×3 input matrix and a 3×3 kernel matrix (with two layers of zero padding rings). Next, we will show that the convolution between the kernel and a larger input matrix can be calculated by overlapping the output matrices of several adjacent smaller submatrices, as explained below.

**Figure 6:**
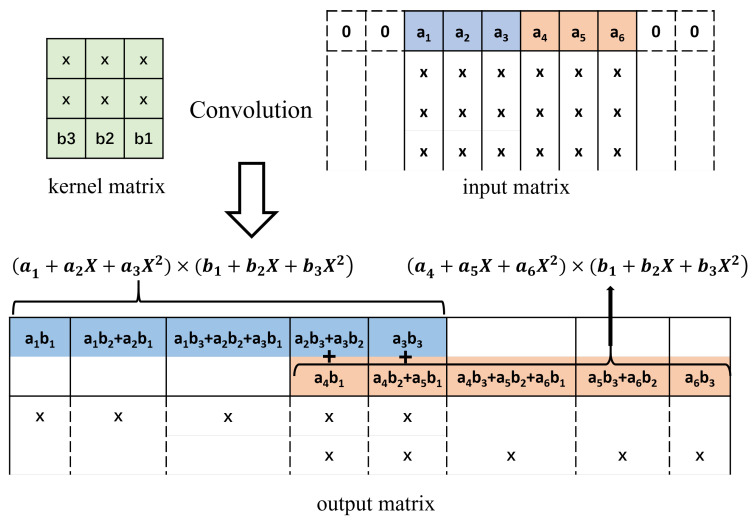
Overlapping one row of the adjacent matrices. Convolution between the first row of kernel matrix (b3,b2,b1) and the first row of input matrix (a1,a2,a3,a4,a5,a6) and the first row of the resulting matrix, where x is the non-relevant elements. The first row of the output matrix is actually the overlapping (overlapped by two elements) between the blue-colored dot-product (b3,b2,b1)(a1,a2,a3) and the red-colored dot-product (b3,b2,b1)(a4,a5,a6).

A 3×3 kernel matrix is to be convolved with an input matrix of dimension 6×6. Without loss of generality, the input matrix is padded with two layers of zeros around the 6×6 area. The convolution after the dot-product calculation is shown in Figure 6.

The resulting output matrix has the dimension of 8×8, of which the elements of the first row are shown in Figure 6. The elements can be split into two subrows: the upper row has 5 elements starting from the first position, and the lower row has another 5 elements, starting from the fourth position. The elements in the upper row correspond to the cross-term of $\left(a_{1}+a_{2}X+a_{3}X^{2}\right)\times \left(b_{1}+b_{2}X+b_{3}X^{2}\right)$, while the elements in the lower row correspond to the cross-terms of $\left(a_{4}+a_{5}X+a_{6}X^{2}\right)\times \left(b_{1}+b_{2}X+b_{3}X^{2}\right)$.

**Figure 7:**
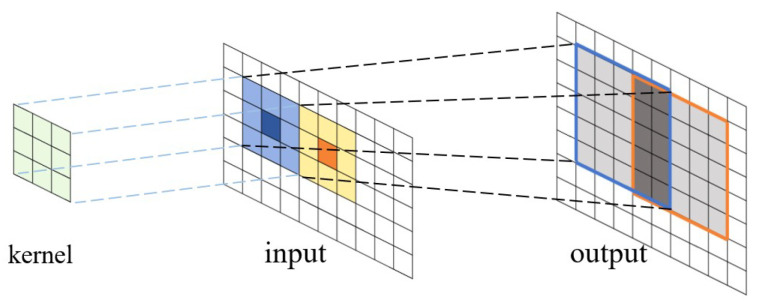
The input tile, convolution kernel, and the output tile of the Karatsuba algorithm. Split the input into a 3×3 tile. Obtain the output size of 5×5. Adjacent output tiles have 2 pixels overlapping.

Graphically, the 8 elements in the first row of the 8×8 resulting matrix are actually the linear overlapping between the upper row and the lower rows, where the last two terms of $\left(a_{1}+a_{2}X+a_{3}X^{2}\right)\times \left(b_{1}+b_{2}X+b_{3}X^{2}\right)$ overlap with the first two elements of $\left(a_{4}+a_{5}X+a_{6}X^{2}\right)\times \left(b_{1}+b_{2}X+b_{3}X^{2}\right)$. As shown in Figure 7.

**Figure 8:**
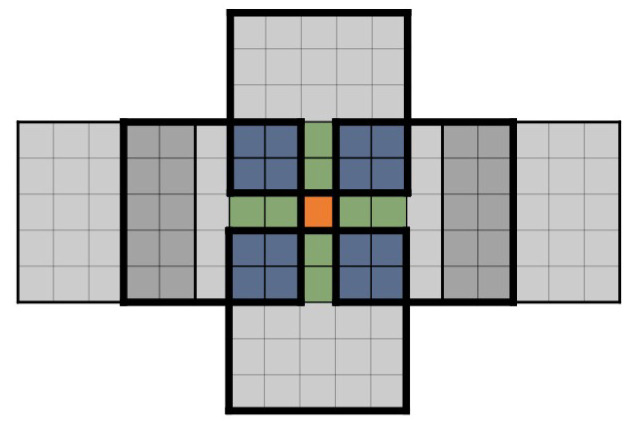
The output is a series of 5×5 matrices. Each output matrix overlaps the adjacent matrices by 2 elements in width.

Similarly, the elements in the first column of the resulting matrix can be decomposed into two subcolumns. The elements in each subcolumn correspond to the cross-terms of multiplication between two 3-term polynomials, and the two subcolumns overlap each other with 2 elements. In fact, when the kernel matrix convolute with a larger matrix, the resulting matrix actually can be decomposed into a series of 5×5 matrix (the result matrix between a 3×3 kernel matrix and a 3×3 input matrix with padding rings, as introduced above) that are overlapped horizontally and vertically, with the overlapping area of 2 elements row-wise and column-wise, as shown in Figure 8.

### 3.4. Divide-and-Conquer Process

The Karatsuba algorithm can be used to split the long operands into any number of segments and reduce the number of multiplications in the product calculation. Therefore, the kernel matrix size does not have to be limited to 3×3, in fact, the kernel matrix size can be any dimension starting from 2×2, while the kernel size of Winograd can only start from 3×3.

**Figure 9:**
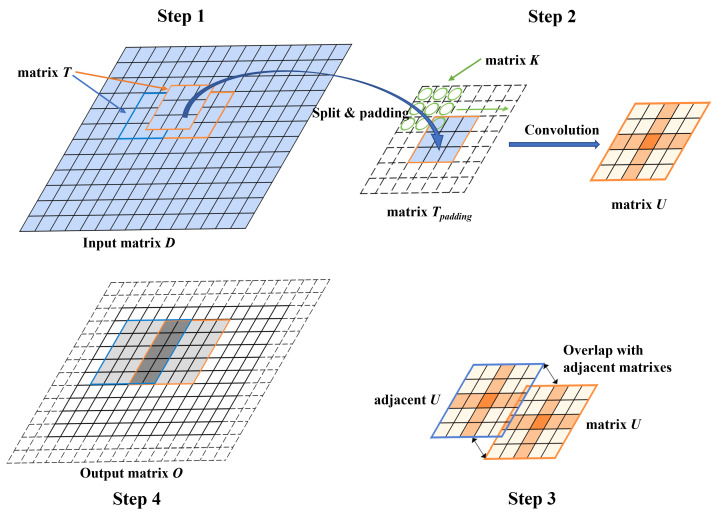
The procedures of the Karatsuba convolution algorithm.

Assuming the kernel matrix K has dimension r×r, and the input matrix D has dimension n×n, the Karatsuba algorithm applied on convolution optimization can be generalized into the following procedures (shown as Figure 9):

(1) Split the input matrix into several abutting tile matrices T; each tile matrix has the same dimension as the kernel matrix, i.e., r×r.

(2) Add r−1 circles of zeros as the padding elements around the tile matrices T, the padded matrix is denoted as $T_{\mathrm{padding}}$. Calculate the convolution between the matrix $T_{\mathrm{padding}}$ and the kernel matrix K, using the Karatsuba algorithm to reduce the multiplication. The resulting matrix *U*, called the overlapping matrix, has the dimension (2r−1)×(2r−1).

(3) The overlapping matrix U will be overlapped with other adjacent U matrices (convoluted between K and other adjacent $T_{\mathrm{padding}}$), the overlapping region is (r−1), i.e., there are (r−1) rows or columns overlapped when two U matrices are overlapped to each other (row-wise or column-wise).

(4) After all matrices U are overlapped, trim the outer r−1 rings from the resulting matrix and obtain the output matrix O. The output matrix O has the dimension, n−r+1, which is the same as the output matrix when performing traditional dot-product convolution.

## 4. Computation Resource Analysis

### 4.1. Elements in the Output Matrix O Are Calculated from Overlapping Adjacent U Matrices

In this section, we estimate the resources, i.e., the number of multiplications and additions, needed for the convolution computation using the Karatsuba algorithm as compared with the Winograd algorithm.

We can use the average number of multiplication/additions needed to calculate each element in the output matrix as the indicator to compare the computation resources between these two algorithms.

**Figure 10:**
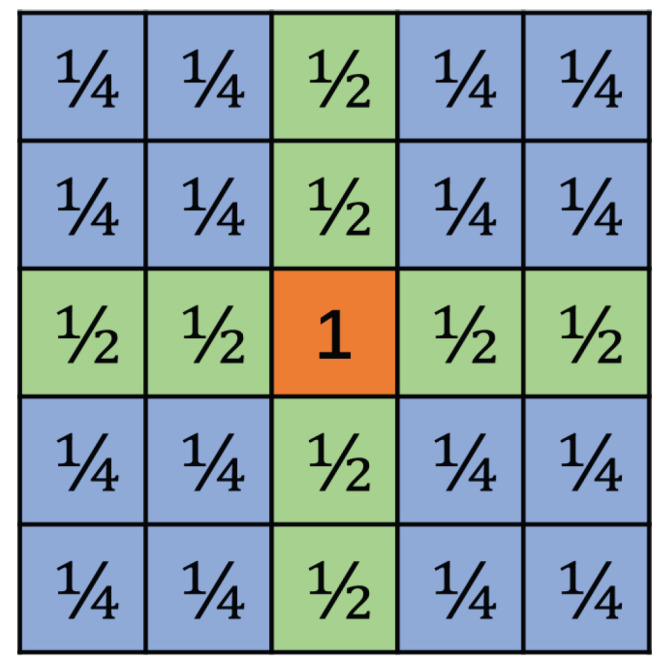
One of the output matrices. The center element is counted as 1; the green elements are counted as ½; and the blue elements are counted as ¼.

As shown in the previous section, for a 3×3 kernel matrix K, the output matrix O is constructed through the overlapping of adjacent 5×5 U matrices. Each element in O is overlapped by different numbers of U matrices. Using one 5×5 matrix U as an example, the center element is not shared by any other 5×5 U matrices; the two elements on the upper, lower, left, and right sides are shared by one neighboring U matrix from up, down, left, and right directions, respectively. The 16 corner elements, four in each corner, are shared by four neighboring U matrices. When we calculate the output elements of this U matrix, the elements shared by two U matrices are counted as ½ elements, and the elements shared by four neighboring U matrices are counted as ¼ elements, as indicated in Figure 10 above.

### 4.2. Effective Element

We introduce the concept of effective elements to measure the number of elements that are computed by one overlapping matrix U. For the 5×5 U matrix, the center element is calculated solely by the same U matrix, while there are 8 elements that are partially calculated by two overlapping U matrices, and 16 elements that are partially calculated by 4 overlapping matrices. Therefore, the effective elements are 9 $\left(1+8\times \frac{1}{2}+16\times \frac{1}{4}=9\right)$.

The effective elements can be conceptually explained as follows. Applying the Karatsuba algorithm to calculate the convolution between a 3×3 kernel matrix and 3×3 input tile matrix and obtain one overlapping matrix U of dimension 5×5, 36 multiplications and 120 additions are needed. However, out of the 25 elements in matrix U, some elements are only partially calculated in this process, and need to wait to overlap with other adjacent U matrices. Meanwhile, the elements in the current U matrix also contribute to the adjacent U matrices. The calculations are equivalent to the calculation of 9 elements, therefore, the number of effective elements is 9.

Furthermore, the overlap between the adjacent matrices U also requires addition. Shown as Figure 10, there are 4 corner areas, and each area has 4 elements. Each corner area is shared by four U matrices, which means each element needs 3 additions. So, four elements cost 12 additions, which are shared by 4 U matrices. As for one particular matrix, it should be counted as 3 additions for each corner. In the same way, the up, down, left, and right sides cost one addition, respectively. Because one matrix has four corners and four sides, 16 additions are required for the overlapping.

To add everything up, one output matrix costs 120 additions for convolution and 16 additions for overlapping. It requires a total of 136 additions for the computation of one output U matrix.

**Figure 11:**
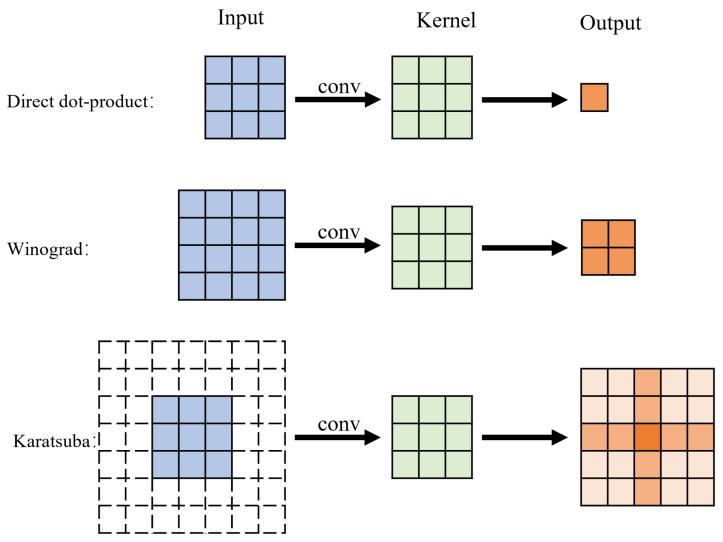
The different input and output sizes of the three algorithms.

The computation resources (number of multiplications and additions) required for Karatsuba-based convolution are compared with the resources needed for the direct dot-product approach and the Winograd-based approach. As discussed above, 5×5 output matrix from Karatsuba has 9 effective elements. In comparison, the output matrices of direct dot-product and Winograd-based convolution do not need to overlap with adjacent matrices, and their effective number of elements is exactly the number of elements in the output matrix, as shown in Figure 11. In order to calculate the average number of computation resources needed to derive one output element, the effective elements should be used. The comparison is listed in Table 2.

It can be observed from the table that the Winograd algorithm and the Karatsuba algorithm both need four multiplications in order to calculate one output element in the output matrix, as compared with 9 multiplications needed by using the direct dot-product. However, while requiring the same number of multiplications, the Karatsuba algorithm needs many fewer additions (around 15) as compared with Winograd (around 19). The Karatsuba algorithm has the potential to outperform the Winograd algorithm in convolutions between larger matrices.

## 5. Hardware Implementation Testing Result

The kernel size of 3 × 3 is widely used in many applications. We therefore use the 3 × 3 kernel to implement the network and compare the hardware resources.

With the 3 × 3 kernel size, the direct dot-product calculation, as well as Winograd and Karatsuba optimizations, can all be constructed from basic operational modules, as shown in Figure 11, namely,

(1) For direct dot-product, the basic module has input matrix size 3 × 3 and output size 1 × 1, or one single output element.

(2) For Winograd optimization, the basic module is F(2 × 2, 3 × 3), i.e., the input matrix is 4 × 4 and output matrix is 2 × 2.

(3) For Karatsuba, the input matrix size is 3 × 3 (padding into 7 × 7 with 2 padding rings), and the output size is 5 × 5.

We first evaluate the hardware resources of these basic convolution modules and then use these modules to construct the complete convolution of full-sized input matrices. The modules are implemented in FPGA platforms. Through the FPGA usage report, the hardware resources used, as well as timing latency, can be compared.

### 5.1. Implementation of Basic Convolution Modules

Direct dot-products, along with the Winograd and Karatsuba algorithms, are first designed in Verilog and then synthesized using Vivado2018 tool. In the FPGA platform, LUT is the basic building block, we can use the number of LUTs in the implementation as an indicator for hardware usage of different approaches.

In order to achieve an LUT-only design, on-board DSP (Digital Signal Processor) has to be prohibited. Therefore, the maximum number of DSPs has been set to zero in our implementation. Vivado2018 also performs sequence partitioning that can segment combinational logic circuits into smaller pipelined blocks. The sequential partition function has also been disabled.

**Figure 12:**
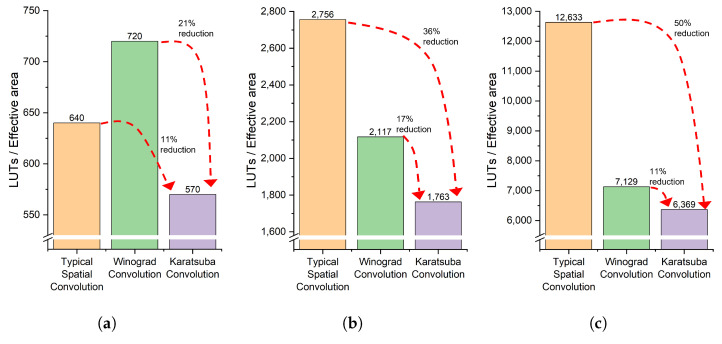
The number of LUTs for convolution of different bit-widths data: (**a**) 8-bit implementation; (**b**) 16-bit implementation; (**c**) 32-bit implementation.

We implement convolution with three different precisions, namely, 8-bit, 16-bit, and 32-bit, for both input matrices as well as the kernel matrix. As discussed in the previous section, their effective output elements numbers for Winograd and Karatsuba optimization are 4 and 9, respectively (while for direct dot-product, the effective element number is 1). So, the total LUT number needs to be divided by its effective elements number, respectively. The results are illustrated in Figure 12. Our experiments were conducted on the Xilinx Artix-7 Series FPGA platform, where all resource utilization results are measured in terms of 6-input LUTs and ESP48E, and all results are reported based on this architecture.

The experiment results are consistent with our algorithmic analysis previously. While the LUT numbers increase almost quadratically with the increase in bit-width (from 8, 16 to 32 bits), under the same bit-width, Karatsuba requires the least number of LUTs. More specifically, in the case of 8-bit Winograd optimization, the saving from multiplication cannot significantly offset the overhead of extra additions; the Winograd needs more LUTs (720) than the direct dot-product (640). Nevertheless, Karatsuba has the lowest LUTs (570). The savings from multiplication will significantly offset the addition overhead for greater bit-widths. Although Karatsuba requires the same number of multiplications as compared with Winograd, because it needs much fewer additions, Karatsuba outperforms Winograd in different bit-widths consistently.

The above results compare the LUT usage with different implementations, where multiplications and additions are computed by LUT logics only, without pipelining the operations. In the Xilinx FPGA platform with DSP blocks, multiplications can also be computed with DSPs (3 DSPs are needed for one multiplication of a 16-bit integer). Alternatively, we enable the DSP usage and pipelining options in the synthesis process and compare the DSP usage along with LUTs. The results of the convolution with a 16-bit width are listed in Table 3.

It can be seen that the DSP(Digital Signal Processor) usage corresponds exactly with the number of multiplications needed for each approach, i.e., 9 (27 DSPs) multiplications needed for direct dot-product, while 4 (12 DSPs) are needed for Winograd and Karatsuba. Besides the DSPs, the remaining LUTs are mainly used for additions and random logic circuits, and Karatsuba has fewer LUTs compared with Winograd.

Because we enable pipelining functions in the HLS, each approach is pipelined automatically by Vivado2018. However, the delay per stage (1 clock per interval) is still the same, and the throughput for each approach is the same.

### 5.2. Implementation of Convolution with Full-Sized Input Matrices

The above basic convolution modules can be used as building blocks to construct a convolution with full-sized input matrices. Instead of comparing the operations for one effective element in the output, as was the case in the previous test, in this test, we implement the convolutions between the 288 × 288 input matrix and 3 × 3 kernel matrix and generate a 286 × 286 output matrix. All three approaches, i.e., the direct dot-product, Winograd, and Karatsuba, generate the output matrices of the same size, therefore, the hardware resource usage can be compared directly and fairly. Without loss of generality, 16-bit-width precision is used for both the input matrix and kernel matrix.

**Figure 13:**
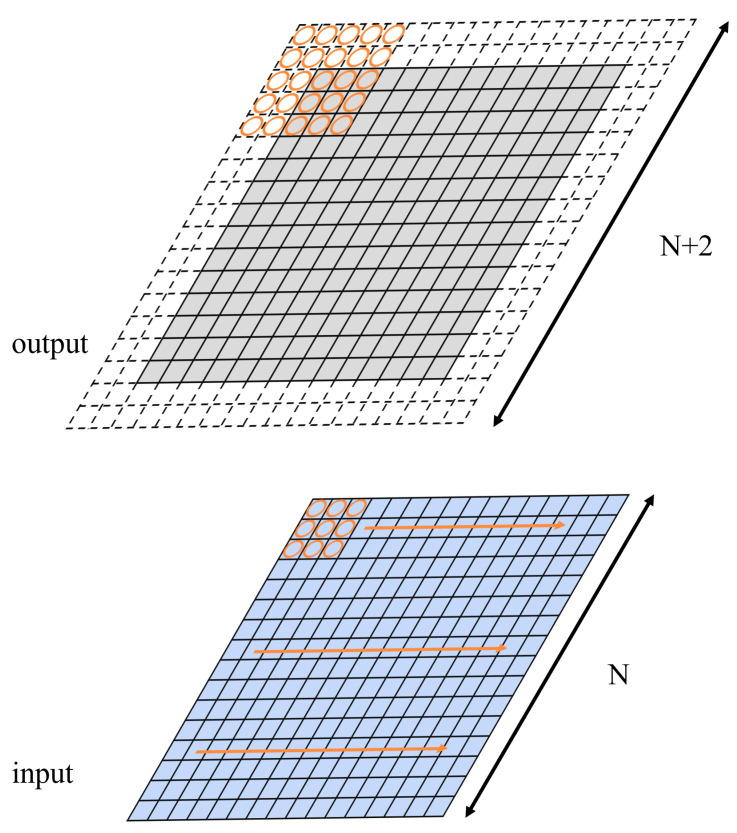
The input and output matrices of the Karatsuba convolution with a 3 × 3 kernel. The orange circle region represents the U matrix from the basic convolution module. The padding rings of the output matrix will later be omitted to generate the output matrix (the gray area).

With the 3 × 3 kernel matrix, two padding rings are needed for the input matrix in Karatsuba optimization, as shown in Figure 13. The padding rings will cost some extra computation (although the elements are all zeros). Nevertheless, for larger-sized input matrices (288 × 288 in this case), this overhead can be neglected.

Because each basic convolution module of direct dot-product, Winograd, and Karatsuba generates a different number of effective elements (i.e., 1, 4, and 9, as shown Figure 11), in order to achieve the same throughput, we implement a computation module (CM) for each of the three approaches that can calculate 36 effective elements at once. Inside each CM, the direct dot-product approach needs 36 basic convolution modules. Winograd optimization needs 9 basic convolution modules (4 effective elements each) and Karatsuba needs 4 basic convolution modules (9 effective elements each).

The FPGA hardware usage report from Xilinx Vivado2018 HLS is listed in Table 4. The results are also consistent with our analysis. While the direct dot-product has the most number of DSPs (972), Winograd and Karatsuba all need the same number, but many fewer DSPs (432). However, Karatsuba needs many fewer LUTs (29526) than Winograd (44454), and the saving from the LUTs is related to the saving of additions in the Karatsuba algorithm, as additions and other random logic functions are implemented by LUTs in FPGA.

It is also interesting to notice that direct dot-product uses the least LUT, because it needs fewer additions at the expense of many more DSPs (for more multiplications). Karatsuba takes a bit longer latency to finish the convolution, but the difference is minimal (2313 vs. 2283).

## 6. Conclusions

In this paper, we propose to use the Karatsuba algorithm to optimize the computation workload of the convolution operation. Compared with the previous commonly used Winograd convolution optimization technique, Karatsuba optimization needs the same number of multiplications but requires a much smaller number of addition operations. The hardware implementation (in FPGA) confirms our analysis and demonstrates that the convolution optimized by the Karatsuba algorithm uses fewer LUTs while achieving similar performance.

## Figures and Tables

**Table 1:** The number of LUTs needed to implement additions and multiplications of operands of different bit-width.

Bit-Width	4-bit	8-bit	16-bit	32-bit
Addition	4	8	16	32
Multiplication	23	61	277	1344

**Table 2:** The theoretical resources comparison of the three algorithms.

Name	Ouput Size	Effective Element Number	Number of Multiplication	Multiplication/ Effective Element	Addition	Addition/ Effective Element
Direct dot- produce	1 × 1	1	9	9	8	8
Winograd	2 × 2	4	16	4	77	19.25
Karatsuba	5 × 5	9	36	4	136	15.11

**Table 3:** The hardware comparison of three kernels using HLS tools.

	Latency	Interval	DSP	DSP/Effective Elements	LUT	LUT/Effective Elements
Direct dot- produce	2	1	27	27	606	606
Winograd	5	1	48	12	5079	1269.75
Karatsuba	7	1	108	12	7492	832.44

**Table 4:** Hardware resources in calculating the convolution between an input matrix of 288 × 288 and a kernel of 3 × 3, generating an output matrix of 286 × 286.

	Latency (cycles)	DSP	LUT
Direct dot-produce	2278	972	16263
Winograd	2283	432	44454
Karatsuba	2313	432	29526

## Data Availability

The original contributions presented in this study are included in the article. Further inquiries can be directed to the corresponding author(s).

## Appendix A

The pseudocode that implements the F(2 × 2, 3 × 3) convolution using the Winograd algorithm is as follows.

The calculation process can be divided into two subroutines, (1) the scalar mode routing, which is named multiplier_conv(), and (2) the vector mode routine.

The scalar mode routine multiplier_conv() actually implements the one-dimensional Winograd algorithm as described in Equation (2). And the vector mode routine actually implements a two-dimensional Winograd algorithm that calls multiplier_conv().

For scalar mode Winograd convolution, *a* has four elements, *b* has three elements, and the output has two elements. According to the above pseudocode, four multiplications and eleven scalar additions are needed. In other words, for the Winograd operation in F(2,3), four multiplications and eleven additions are required in the routine.

In the vector mode subroutine, *a* has four vectors, each of which has four elements, which means *a* is actually a 4 × 4 matrix. Similarly, *b* has three vectors, and each vector has 3 elements. The output has 2 vectors, and each vector has 2 elements.

From the vector mode routine, we can find that there are four convolutional multiplications multiplier_conv(), four add_vector4(), four add_vector2(), and three add_vector3() in total.

For each convolutional multiplication multiplier_conv(), it actually encapsulates the scalar mode subroutine. The input length is three elements, and the output length is two elements. As each convolutional multiplication costs four multiplications and eleven additions, four convolutional multiplications multiplier_conv() can cost sixteen scalar multiplications and forty-four scalar additions in total.

For add_vector2() (i.e., addition of two vectors, and each vector has 2 elements), as the size of the input vector is two (the output size of the convolutional multiplication is 2), each vector2 addition costs two scalar additions, and four vector2 additions cost eight scalar additions in total.

For add_vector3() (i.e., addition of two vectors, each vector has 3 elements), as the size of the input vector *b* is three, each vector3 addition costs three scalar additions, and three vector3 additions cost nine scalar additions in total.

For add_vector4() (i.e., addition of two vectors, and each vector has 4 elements), as the size of the input vector *a* is four, each vector4 addition costs four scalar additions, and four vector4 additions cost sixteen scalar additions in total.

Therefore, the vector mode Winograd costs sixteen multiplications and seventy-seven additions (forty-four from convolutional multiplications, eight from add_vector2(), nine from add_vector3(), and sixteen from add_vector4()).

**Algorithm A1** The pseudocode for the Winograd convolution algorithm	
1: //the scaler mode Winograd subroutine 2: **function** multiplier_conv(a[1:4], b[1:3]) 3: a1 = a[1]; a2 = a[2]; a3 = a[3]; a4 = a[4]; 4: b1 = b[1]; b2 = b[2]; b3 = b[3]; 5: add_1 = add_scalar(a1,negative(a3)) 6: add_2 = add_scalar(a2,a3) 7: add_3 = add_scalar(a3,negative(a2)) 8: add_4 = add_scalar(a2,negative(a4)) 9: add_5 = add_scalar(b1,negative(b3)) 10: ** ** 11: add_6 = add_scalar(add_5,b2) 12: add_7 = add_scalar(add_5,negative(b2)) 13: ** ** 14: srl_1 = shift_right(add_2) 15: srl_2 = shift_right(add_3) 16: ** ** 17: mul_0 = multiplier_scalar(add_1,b1) 18: mul_3 = multiplier_scalar(add_4,b3) 19: ** ** 20: mul_1 = multiplier_scalar(srl_1,add_6) 21: mul_2 = multiplier_scalar(srl_2,add_7) 22: ** ** 23: add_8 = add_scalar(mul_0,mul_1) 24: add_9 = add_scalar(mul_1,negative(mul_3)) 25: ** ** 26: add_10 = add_scalar(mul_0,mul_1) 27: add_11 = add_scalar(mul_1,negative(mul_3)) 28: c[1] = add_10 29: c[2] = add_11 30: **return** c[1:2] 31: **end function** 32: ** ** 33: ** **	34: //the vector mode Winograd subroutine 35: **function** Convolution(a[1:4][1:4], b[1:3][1:3]) 36: //each element here is a subvector 37: a1 = a[1][1:4]; a2 = a[2][1:4]; 38: a3 = a[3][1:4]; a4 = a[4][1:4]; 39: b1 = b[1][1:3]; b2 = b[2][1:3]; b3 = b[3][1:3]; 40: add_1 = add_vector4(a1,negative(a3)) 41: add_2 = add_vector4(a2,a3) 42: add_3 = add_vector4(a3,negative(a2)) 43: add_4 = add_vector4(a2,negative(a4)) 44: add_5 = add_vector3(b1,negative(b3)) 45: ** ** 46: add_6 = add_vector3(add_5,b2) 47: add_7 = add_vector3(add_5,negative(b2)) 48: ** ** 49: srl_1 = shift_right(add_2) 50: srl_2 = shift_right(add_3) 51: ** ** 52: mul_0 = multiplier_conv(add_1,b1) 53: mul_3 = multiplier_conv(add_4,b3) 54: ** ** 55: mul_1 = multiplier_conv(srl_1,add_6) 56: mul_2 = multiplier_conv(srl_2,add_7) 57: ** ** 58: add_8 = add_vector2(mul_0,mul_1) 59: add_9 = add_vector2(mul_1,negative(mul_3)) 60: ** ** 61: add_10 = add_vector2(mul_0,mul_1) 62: add_11 = add_vector2(mul_1,negative(mul_3)) 63: c[1] = add_10 64: c[2] = add_11 65: **return** c[1:2] 66: **end function**

## Appendix B

Similar to the pseudocode in the Winograd algorithm, we also introduce two subroutines to implement the convolution operation using the Karatsuba algorithm, namely, (1) the scalar mode subroutine and (2) the vector mode subroutine.

In the scalar mode subroutine, both inputs a and b have three elements. The output has five elements. From the pseudocode, we can find that there are six scalar multiplications and twelve scalar additions. This indicates that the scalar mode routine needs six multipliers and twelve adders.

In the vector mode subroutine, both input a and b also have three subvectors. Each subvector has three elements, just like the input of the scalar mode of Karatsuba. The output of the vector mode Karatsuba convolution has five subvectors, and each subvector has five scalar elements.

The scalar mode subroutine actually implements a one-dimensional Karatsuba algorithm like Equation (13). And the vector mode subroutine actually implements a two-dimensional Karatsuba algorithm. The operations of the scalar mode subroutine are encapsulated into the function multiplier_conv(). Inside the vector mode subroutine, the call of multiplier_conv() performs the one-dimensional Karatsuba algorithm where the vectors are the operands.

In the vector mode pseudocode, six convolutional multiplications, six add_vector3(), and six add_vector5() are needed in total.

For each convolutional multiplication multiplier_conv(), it is actually the encapsulation of a scalar mode subroutine, which we illustrated before. The input length is three scalars, and the output length is five scalars. As each convolutional multiplication multiplier_conv() costs six scalar multiplications and twelve additions, six convolutional multiplications multiplier_conv() can cost thirty-six scalar multiplications and seventy-two scalar additions in total.

For add_vector3() (i.e., addition of two vectors, and each vector has 3 elements), as the size of the input vector is three, each vector3 addition costs three scalar additions, and six vector3 additions cost eighteen scalar additions in total.

For add_vector5() (i.e., addition of two vectors, and each vector has 5 elements), as the size of the input vector is five (the size of the output vector of multiplier_conv() is five), each vector5 addition costs five scalar additions, and six vector5 additions cost thirty scalar additions in total.

So, the vector mode Karatsuba costs 36 multiplications and 120 additions (72 from convolutional multiplications, 18 from add_vector3(), and 30 from add_vector5()).

**Algorithm A2** The pseudocode for the Karatsuba convolution algorithm	
1: //the scaler mode Karatsuba subroutine 2: **function** multiplier_conv(a[1:3], b[1:3]) 3: a1 = a[1]; a2 = a[2]; a3 = a[3]; 4: b1 = b[1]; b2 = b[2]; b3 = b[3]; 5: mul_1 = multiplier_scalar(a1,b1) 6: mul_2 = multiplier_scalar(a2,b2) 7: mul_3 = multiplier_scalar(a3,b3) 8: ** ** 9: add_1 = add_scalar(a1,a2) 10: add_2 = add_scalar(b1,b2) 11: add_3 = add_scalar(a2,a3) 12: add_4 = add_scalar(b2,b3) 13: ** ** 14: add_5 = add_scalar(add_1,a3) 15: add_6 = add_scalar(add_2,b3) 16: ** ** 17: mul_4 = multiplier_scalar(add_1,add_2) 18: mul_5 = multiplier_scalar(add_3,add_4) 19: mul_6 = multiplier_scalar(add_5,add_6) 20: ** ** 21: add_7 = add_scalar(mul_4,negative(mul_2)) 22: add_8 = add_scalar(mul_5,negative(mul_2)) 23: add_9 = add_scalar(add_7,negative(mul_1)) 24: add_10 = add_scalar(add_8,negative(mul_3)) 25: add_11 = add_scalar(mul_6,negative(add_7)) 26: add_12 = add_scalar(add_11,negative(add_8)) 27: ** ** 28: c[1] = mul_1 29: c[2] = add_9 30: c[3] = add_12 31: c[4] = add_10 32: c[5] = mul_3 33: **return** c[1:5] 34: **end function** 35: ** **	36: //the vector mode Karatsuba subroutine 37: **function** Karatsuba_Conv(a[1:3][1:3], b[1:3][1:3]) 38: a1 = a[1][1:3]; a2 = a[2][1:3]; a3 = a[3][1:3]; 39: b1 = b[1][1:3]; b2 = b[2][1:3]; b3 = b[3][1:3]; 40: mul_1 = multiplier_conv(a1,b1) 41: mul_2 = multiplier_conv(a2,b2) 42: mul_3 = multiplier_conv(a3,b3) 43: ** ** 44: add_1 = add_vector3(a1,a2) 45: add_2 = add_vector3(b1,b2) 46: add_3 = add_vector3(a2,a3) 47: add_4 = add_vector3(b2,b3) 48: ** ** 49: add_5 = add_vector3(add_1,a3) 50: add_6 = add_vector3(add_2,b3) 51: ** ** 52: mul_4 = multiplier_conv(add_1,add_2) 53: mul_5 = multiplier_conv(add_3,add_4) 54: mul_6 = multiplier_conv(add_5,add_6) 55: ** ** 56: add_7 = add_vector5(mul_4,negative(mul_2)) 57: add_8 = add_vector5(mul_5,negative(mul_2)) 58: add_9 = add_vector5(add_7,negative(mul_1)) 59: add_10 = add_vector5(add_8,negative(mul_3)) 60: add_11 = add_vector5(mul_6,negative(add_7)) 61: add_12 = add_vector5(add_11,negative(add_8)) 62: ** ** 63: c[1] = mul_1 64: c[2] = add_9 65: c[3] = add_12 66: c[4] = add_10 67: c[5] = mul_3 68: **return** c[1:5] 69: **end function**
